# Complete Genome Sequence of *Ralstonia pickettii* DTP0602, a 2,4,6-Trichlorophenol Degrader

**DOI:** 10.1128/genomeA.00903-13

**Published:** 2013-10-31

**Authors:** Yoshiyuki Ohtsubo, Nobuyuki Fujita, Yuji Nagata, Masataka Tsuda, Tomohiro Iwasaki, Takashi Hatta

**Affiliations:** Department of Environmental Life Sciences, Graduate School of Life Sciences, Tohoku University, Sendai, Japana; Biological Resource Center, National Institute of Technology and Evaluation, Shibuya-ku, Tokyo, Japanb; Department of Biomedical Engineering, Okayama University of Science, Kita-ku, Okayama, Japanc

## Abstract

*Ralstonia pickettii* strain DTP0602 utilizes 2,4,6-trichlorophenol as its sole carbon and energy source. Here, we report the complete genome sequence of strain DTP0602, which comprises three chromosomes and no plasmids. We also found that the two *had* gene clusters responsible for the degradation of 2,4,6-trichlorophenol are located on the 2.9-Mb chromosome 2.

## GENOME ANNOUNCEMENT

2,4,6-Trichlorophenol (2,4,6-TCP), which is widely used as a biocide and preservative, is considered a priority environmental pollutant worldwide (1). *Ralstonia pickettii* strain DTP0602, which was isolated from a soil sample in Okayama, Japan, utilizes 2,4,6-TCP as its sole carbon and energy source (2). In this strain, two gene clusters, *hadXABC* and *hadYD*, are involved in the conversion of 2,4,6-TCP to 3-oxoadipate (3), where *hadXABC* and *hadYD* are regulated by *hadR* and *hadS*, respectively (3, 4).

The DTP0602 genome was sequenced using the 454 GS-FLX Titanium (Roche) and GAIIx systems (Illumina). A fragment library and a paired-end library were constructed for 454 GS-FLX sequencing, which obtained 694,430 reads and 272 Mb of data. We conducted 151-bp paired-end sequencing with Illumina GAIIx, which obtained 6,437,388 reads and 972 Mb of data. The reads obtained using both systems were assembled using Newbler version 2.6 (Roche), which produced 508 contigs and 24 scaffolds. The finishing was facilitated using our two original computer programs, GenoFinisher and AceFileViewer (http://www.ige.tohoku.ac.jp/joho/gf_e/). To determine the order of the scaffolds based on *in silico* analyses, GenoFinisher was used to draw contig graphs, while AceFileViewer was used to find and analyze unique variable bases, which are potentially useful for identifying adjacent scaffolds. For three kinds of repeats that were responsible for six, three, and two scaffold gaps, combinatorial PCR experiments were carried out to determine the scaffold adjacency. The DNA sequences of the scaffold gaps were determined using AceFileViewer.

Most of the gaps in the scaffolds were closed by *in silico* analyses using AceFileViewer, which closed all 97 repeat-induced gaps and seven out of 10 true gaps. The DNA sequences of the three remaining true gaps were determined by PCR and subsequent sequencing of the PCR products. The finished sequence was checked by FinishChecker, which is a subtool in GenoFinisher.

The sequence was annotated by the NCBI Prokaryotic Genomes Automatic Annotation Pipeline, and the start codon positions were manually curated using the annotation support tool in GenomeMatcher (5).

The complete sequence of the DTP0602 genome comprises three circular chromosomes (Chr), Chr 1 (4,499,145 bp, 66.12% GC content, 4,410 open reading frames [ORFs]), Chr 2 (2,889,590 bp, GC 66.49%, 2,654 ORFs), and Chr 3 (737,115-bp, GC 62.37%, 685 ORFs). Chr 1, Chr 2, and Chr 3, have three, two, and one copies of rRNA operons, respectively. Fifty-six, nine, and two tRNA genes are located on Chr 1, Chr 2, and Chr 3, respectively. The two *had* gene clusters involved in 2,4,6-TCP degradation are located on Chr 2. The locations of the clusters are unique to DTP0602, although an *hadRXABC*-related gene cluster is found in the large plasmid of *Cupriavidus necator* strain N-1 (6) and in Chr 1 of *C. necator* strain JMP134 (7). The complete sequence of DTP0602 will facilitate further studies of the acquisition of chlorophenol degradation genes, such as *had* and *tcp* (8).

### Nucleotide sequence accession numbers. 

The genome sequence of *R. pickettii* DTP0602 has been deposited in the NCBI under accession no. CP006667, CP006668, and CP006669 for Chr 1, Chr 2, and Chr 3, respectively.
